# Interaction of cyanobacteria and microplastics polystyrene spiked with pharmaceutical drug-paracetamol

**DOI:** 10.1038/s41598-025-01507-z

**Published:** 2025-07-16

**Authors:** Rubina Yasmine, Haleema Naaz, Razique Anwer, Nikhat Manzoor, Tasneem Fatma

**Affiliations:** 1 Department of Biosciences, Jamia Millia Islamia, New Delhi, India; 2 Department of Pathology, College of Medicine, Imam Mohammad Ibn Saud Islamic University (IMSIU), Riyadh, Saudi Arabia

**Keywords:** Microplastics, Polystyrene, Paracetamol, *Nostoc*, Oxidative stress, Ecology, Environmental sciences

## Abstract

The microplastics polymer—polystyrene, polyethylene, polyvinyl chloride, etc. are now recognized as potent threats to the aquatic system due to the Trojan horse effect i.e., they adsorb other pollutants such as pharmaceutical drugs, organic solvents, metals etc. and act as a vector or carrier. Polystyrene (PS) is one of the most usable plastics worldwide that abundantly contaminates the aquatic body. However, to date, only a few studies have focused on the eco-toxic effects of polystyrene in combination with other pollutants. Therefore, in the present study, the effect of polystyrene (pristine) and spiked with the emerging pollutant paracetamol (PCM) was studied on cyanobacterium- *Nostoc muscorum.* PS, spiked with paracetamol exhibited a higher adverse effect on the growth and biochemical constituents. Fluorescence intensities of confocal images of the samples decreased with increasing toxic effect of polystyrene when spiked with paracetamol. Increased laccase and esterase activity also indicated the degradation potential of *Nostoc muscorum.* The findings of present work suggested PS (Pristine and spiked with PCM) toxicity on primary producer of ecosystem and role of cyanobacterial degrading enzymes in bioremediation of PS. Therefore, it is better to “nip in the bud” the plastic pollution rather than to face a great environmental threat.

## Introduction

Microplastics are contaminants of emerging concern, due to their worldwide occurrence, especially in aquatic environments. Microplastics are very small-sized (less than 5 mm) and inert particles^1^. Based on production, microplastics (MPs) can be classified into two categories: primary, which is intentionally manufactured for commercial purposes such as in cosmetics and resin particles, and secondary MPs which are formed accidentally due to the degradation of large-sized plastics^2^. Large-sized plastic and its materials play an important role in daily life. During the COVID-19 pandemic, the consumption of plastics (face masks, personal protective equipment, gloves, sanitizer bottles) increased with poor disposal^3^. The improper disposal of plastics increased the production of secondary microplastics that were consequently released in higher amounts in aquatic ecosystems^4^. People infected with the coronavirus experienced high fever with body aches, and paracetamol (also known as acetaminophen) helped to relieve symptoms associated with coronavirus, which ultimately increased the consumption of paracetamol^5^. The overconsumption of paracetamol during the COVID-19 pandemic increased the level of paracetamol traces in aquatic bodies^6^. Paracetamol is not only found in sewage water, and surface water but also in drinking water. Now, microplastics and paracetamol co-contaminate the aquatic bodies and have emerged as prominent concern as collectively they exert higher adverse effects on aquatic organisms.

In aquatic bodies, cyanobacteria (photosynthetic prokaryotes) are predominantly found as a producer and play a vital role in the aquatic food chain^7^. With the presence of microplastics, pharmaceutical drug traces, and other anthropogenic contaminants in aquatic system, ultimately disrupt the growth and survival of cyanobacteria (aquatic primary producer) and other higher trophic-level organisms that ultimately alter ecological balance^8^. Polystyrene (PS) key component is styrene which poses health risks through ingestion, inhalation, and chemical leaching, leading to inflammation, respiratory issues, and endocrine disruption and poses potential carcinogen linked to leukaemia, human breast epithelial cells and human breast cancer cells^9,10^. Surface-functionalized PS nanoparticles enter inside A549 lung carcinoma cells, that decreased cell viability and enhanced genotoxicity due to micronuclei formation and ROS production^11^.In animals PS micro- and nanoparticles jointly induced intestinal barrier dysfunction via reactive oxygen species (ROS)-mediated epithelial cell apoptosis concomitant with their dynamic bio distribution in the blood and various organs, such as the spleen, kidney, heart, brain, lungs and liver^12^.

Therefore, during the present investigation, the effect of microplastics i.e., polystyrene and pharmaceutical drugs (i.e., paracetamol) was observed on cyanobacterium *Nostoc muscorum* with respect to growth biochemical constituents.

## Materials and methods

### Chemicals, reagents, culture maintenance

All Chemicals and polystyrene beads used in this study were of analytical grade and were purchased from Sigma Aldrich, USA. The reagents and buffers were prepared in double-distilled water. *Nostoc muscorum* NCCU-442 was grown in sterilized BG-11 medium without nitrogen source in 250-ml flasks at 27 ± 2 °C with 12:12 light: dark condition under cool white fluorescent tubes at 25-µmol photons min^–1^ light intensity. To dissolve polystyrene (0.05 mg), its beads were dissolved in 0.4% acetone and added to the culture medium directly.

### Growth and biochemical analysis of the test organism exposed to polystyrene and Paracetamol

For determining the LC_50_ Values, *Nostoc muscorum* was exposed to different concentrations of acetone (0.1%, 0.2%, 0.3%, 0.4%, 0.5%, 0.6%, 0.7%, 0.8%, 0.9%, 1.0%), polystyrene (10%, 20%, 30%, 40%, 50%, 60%, 70%, 80%, 90%, 100%) and paracetamol (10%, 20%, 30%, 40%, 50%, 60%, 70%, 80%, 90%, 100%) and growth was evaluated for 24 days in 200 mL culture medium. After determining the growth of the test organism, the % reduction was evaluated, and the LC_50_ was calculated through probit analysis^13^.

Further, the LC_50_ value of polystyrene, paracetamol, and acetone was used for setting the main experiment to understand the effect of polystyrene (pristine and spiked with paracetamol) on the growth, biochemical constituents (pigments protein, malondialdehyde, H_2_O_2_, antioxidant enzyme non-enzymatic antioxidant) and electrolyte leakage in *N. muscorum*. The experiments were set as follows and analyzed on every 3rd day till 24 days.

**Table:** 

SET-I	Nostoc muscorum in BG11medium
SET-II	*Nostoc muscorum* in BG11medium + Acetone
SET-III	*Nostoc muscorum* in BG11medium + Polystyrene
SET-IV	*Nostoc muscorum* in BG11medium + Paracetamol
SET-V	*Nostoc muscorum* in BG11medium+ Polystyrene + Paracetamol

The content of chlorophyll was determined by the modified method of^14^. 10 mL of 95% methanol was added to 5 mg of dried biomass in a test tube and incubated at 65 °C for 30 min in a water bath. Then, centrifuged at 5000 rpm for 10 min. Pellets were discarded and absorbance of the supernatant was taken at 650 nm and 660 nm against 95% methanol as blank.

Carotenoid content was estimated according to the method given by^15^. Biomass (50 mg) was taken in the test tube to which 5 mL of 80% acetone was added. The content was then homogenized and centrifuged at 3000 rpm for 10 min. The absorbance of the supernatant was recorded at 450 nm against 80% acetone as blank.

The protein content was determined according to the method given by^16^. Biomass (50 mg) was homogenized in 5 mL of extraction buffer and centrifuged at 8000 rpm for 20 min. In supernatant, reagent-A (prepared by adding 1 mL freshly prepared 1% Na-K tartrate solution containing 0.5% CuSO_4_ into 50 mL 2% Na_2_CO_3_ solution) was added and incubated for 10 min. After which, 0.5 mL of reagent-B (Folin reagent) was added and kept in the dark for 30 min to develop the colour that indicates the presence of proteins. A standard solution of bovine serum albumin (BSA) was prepared in different concentrations for quantification of total protein and absorbance was taken at 650 nm.

The oxidative damage was analysed by estimating MDA and hydrogen peroxide content. The MDA content was measured by the method given by^17^. Cells were homogenized in 1% Trichloroacetic acid (TCA) and centrifuged at 10,000 rpm for 15 min. The supernatant was re-suspended in 0.5% thiobarbituric acid (TBA) in 20% TCA solution and then incubated in a water bath at 95 °C for 30 min and cooled under running water. Absorbance was taken at 532 nm and 600 nm and MDA was estimated using extinction coefficient (155 mM-1 cm-1).

Hydrogen peroxide content was measured by the method given by^18^. Fresh biomass (50 mg) was homogenized in 1.5 mL sodium phosphate buffer (50 mM, pH 6.5) and centrifuged at 10,000 rpm for 15 min. To determine the hydrogen peroxide, 1 mL of supernatant was mixed with 4 mL of 0.1% titanium sulphate containing 20% sulphuric acid. After the development of the yellow colour, absorbance was taken at 410 nm hydrogen peroxide was estimated using extinction coefficient (0.28 µmol cm^–1^).

Electrolyte leakage was estimated according to the method given by^19^. The 50 mg biomass (control/ PS exposed) was washed with washing buffer (EDTA buffer-pH 7.6) and then with double distilled water. After washing, the sample was dissolved in 30 mL of double distilled water and incubated for 24 h at 30 °C. Then, the sample was centrifuged at 5000 rpm for 10 min, discarded the pellets, and took the initial electrolyte conductivity (EC_1_) of the supernatant on a digital conductivity meter (CC-607, century, India). After measuring the EC_1_, the samples were incubated in a water bath at 100 °C for 15 min, at this stage, all the electrolytes were released and then the sample was cooled under running water and centrifuged at 5000 rpm for 10 min and thereafter discarded the pellets and took the final electrolyte conductivity (EC_1_) of the supernatant.

SOD activity was determined by the method of^20^. Fresh biomass (0.05 gm) was homogenized in 2 mL of phosphate buffer (0.5 M, pH 7.5) and centrifuged at 15,000 g at 4 °C for 15 min. The 3 mL reaction mixture contained 2.9 mL potassium phosphate (50 mM, pH 7.8) containing 0.025% Triton-X 100, 1.17 µM riboflavin, 168 µM NBT, 10 mM methionine and 100 µL extract. Blank contained everything except the enzyme extract. Both the reaction mixture and blank were incubated for 15 min. The photo-reduction of nitro blue tetrazolium chloride (NBT) resulted in the formation of purple formazan, measured at 560 nm. 1U of SOD activity is the amount of enzyme that causes 50% inhibition of the photo-reduction of NBT i.e., formazan formation. CAT activity was determined by the method of^21^. Fresh biomass (0.05 gm) was homogenized in 2mL of phosphate buffer (0.5 M, pH 7.5) and centrifuged at 10,000 rpm at 4 °C for 15 min. Reaction mixture (2.0 mL) contained 0.1 mL of enzyme extract, 1.6 mL phosphate buffer (pH 7.3), 0.1 ml of EDTA (3 mM) and 0.2 mL of H_2_O_2_ (0.3%) and was incubated for 3 min. And absorbance was taken at 240 nm. Catalase activity was calculated by taking ℇ _240_ as 43.6 M^–1^.cm^–1^ against a blank of the same reaction mixture except for hydrogen peroxide. APX activity was determined by the protocol of^22^. Fresh biomass (0.05 gm) was homogenized in 2 mL of phosphate buffer (0.5 M, pH 7.5) and centrifuged at 10,000 rpm at 4 °C for 15 min. Reaction mixture was prepared by mixing 1 mL phosphate buffer (pH 7.3), 1 mL ascorbate (5 mM), 0.1 mL EDTA, and 0.2 mL hydrogen peroxide (0.3%) and 1 mL enzyme extract. Absorbance was taken at 290 nm against blank. The activity of APX was calculated using an extinction coefficient of 2.8 mM^–1^cm^–1^.

Proline content was determined by the method of^23^. Dried biomass (0.05 g) was homogenized in 10 mL of methanol (80%). The homogenate was then filtered with Whatman filter paper to obtain the methanolic extract. Distilled water (5 mL) and Folin Ciocalteu’s reagent (0.5 mL) were added to the methanolic extract (40 µL) and kept for 5 min. Then, 1.2 mL sodium carbonate (20%) was added and the volume of the test sample was adjusted to 10 mL with distilled water and allowed to stand in dark at room temperature for 60 min., Absorbance was recorded at 750 nm.

### Degradative enzymes activity (pristine and spiked with paracetamol)

For the evaluation of the degradation of polystyrene and paracetamol, degradative enzymes (Laccase and esterase) activity was analyzed. The modified^24^ technique, which is based on the oxidation of ABTS, was used to measure the activity of laccase. 500 µl of the culture supernatant was added to the assay mixture (300 µl of 2 mM ABTS, 200 µl of 100 mM citrate buffer (pH 4.0) and incubated for 10 min. Activity of enzymes was checked by the oxidation of ABTS. The oxidation of ABTS was monitored by taking the absorbance at 420 nm. The enzyme activity (UL^–1^) was calculated by taking ℇ420 as 3.6 × 10^4^ M^–1^ cm^–1^^25^. The hydrolysis of 4-nitrophenyl acetate technique was used to measure the released paranitrophenol to assess the esterase activity^26^. The assay mixture containing 500 µl potassium phosphate buffer (50 mM, pH 7.0), 100 100 µl 4-nitrophenyl acetate (25 mM) and 250 µl culture supernatant was diluted to 150 µl of distilled water and incubate at 37 °C for 30 min. The para-nitrophenyl acetate substrate was hydrolysed to produce acetic acid and para-nitrophenol. The enzyme activity was measured using a 400 nm absorbance against potassium phosphate buffer, with UL-1 being the unit of enzyme release per minute at 37 °C.

### Morphological study of Nostoc muscorum under exposure of polystyrene (pristine and spiked with paracetamol)

Confocal microscopy, scanning electron microscopy, and transmission electron microscopy were done for morphological study.

Confocal microscopy was done to check the effect of acetone, polystyrene, and paracetamol on *Nostoc muscorum* growth measured as fluorescence. For fixation of homogenised samples 1mL culture from all five sets, 2.5% of 0.2 M glutaraldehyde phosphate buffer having pH 7.0 was added for fixation and kept for 3 h. Then, they were washed three times with the same buffer and stored in 5mL phosphate buffer at 4 °C. From this, a sample drop was placed on the slide and slowly covered with a glass slip. Slide was observed with a X63 1.4 numerical aperture and apochromatic oil immersion objective lens under an excitation beam with a wavelength of 568 nm by an Argon-Krypton laser beam^27^. A fluorescent image of living cells was captured. The cyanobacterial photosynthetic pigments, chlorophyll and phycobilins, have an inherent fluorescence at 543 and 633 nm excitation and 590–800 nm emission and need no labelling for visualization. Transmission micro photo of the same sample was taken that showed the images of both living and non-living cells as dark object in white background^28^. The comparison of the fluorescent image with the transmission image reflects the toxic nature of the exposed cells, if any.

To check the surface morphology, scanning electron microscopy of the cells also done. The harvested cells from all sets were washed and then fixed in the fixing buffer (100 mM, pH 7.4 phosphate buffer that contain 2.5% glutaraldehyde and 2% paraformaldehyde) and kept at 4 °C for 12 h. Before scanning the samples ionized by ion soutter on the metal stub for 20 min. Following the application of a gold coating, the samples were placed under vacuum and subjected to scanning micrography using a SEM (ZEISS EVO 18) at magnifications ranging from 50 to 15,000 x, to obtain micrographs.

A transmission electron microscope is used to investigate the ultrastructure. The freshly harvested biomass was subjected to centrifuging for 10 min at 6000 rpm, and the algal pellets were resuspended in 0.01 M phosphate buffer (pH 6.8) containing 2% glutaraldehyde and the content was fixed at 4 °C for 24 h. After that, the samples were centrifuged and the fixed algal pellets were directly mixed with phosphate buffer containing 4% agar. A one mm^3^ piece of solidified agar was rinsed in phosphate buffer for 1 h, then osmium fixation was continued at 4 °C for 2 h. The samples were washed, dehydrated, and fixed in epoxy resin. The sections were viewed using a Transmission Electron Microscope (TECNAI 200 Kv).

Statistical analysis was done by using GraphPad Prism Version 8.0.1 (244). All data were evaluated statistically and represented as mean ± standard deviation of three independent replicates. Means were compared by t-test and one-way ANOVA followed by least significant differences (LSD) test at levels *P* < 0.05.

## Result and discussion

**Fig. 1:**
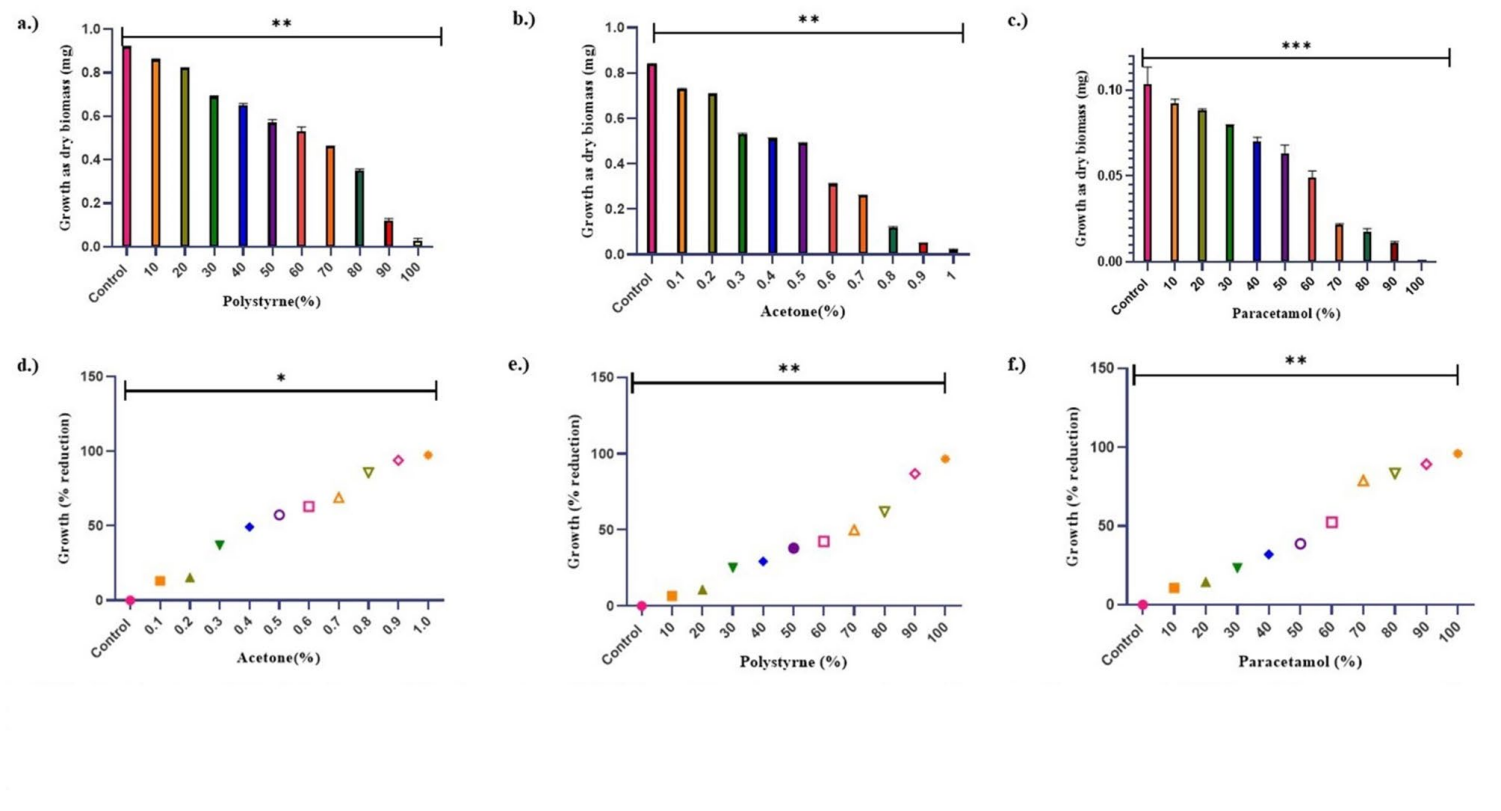
(a–c) Effect of Acetone, Polystyrene and Paracetamol on growth as dry biomass. (d–f) Determination of LC_50_ for Acetone, Polystyrene and Paracetamol. All the values are the means of three replicates with standard deviation and asterisks (*) indicating the level of significance at *P* < 0.05.

Acetone (polystyrene solvent) inhibited cyanobacterial growth that was directly proportional to solvent concentration and exhibited LC_50_ value of 0.4% acetone (Fig. 1b and d). Microplastics polystyrene was dissolved in LC_50_ value of solvent acetone (0.4%) and cyanobacteria was allowed to grow in polystyrene (dissolved in acetone) for determination of its LC_50_ value. An increase in polystyrene dose resulted in a gradual decrease in growth and LC_50_ value was obtained at 6 mM (Fig. 1a and e). Paracetamol (dissolved in water) also exhibited dose-dependent growth reduction (measured as dry biomass) in the test organism and its LC_50_ value was determined at 3.5 mM (Fig. 1c and f).

### Effect of PS (pristine and spiked with PCM) on the growth of Nostoc muscorum

Then, the experiment was planned and executed to observe whether the spiking of microplastic polystyrene with paracetamol exerts higher toxicity on an aquatic prokaryotic photosynthetic organism *Nostoc muscorum* or not. A total five experimental treatments were given to the test organism and all sets were allowed to grow in similar conditions. SET I—Control, SET II—Acetone, SET III—Polystyrene, SET IV—Paracetamol, SET V—Polystyrene + Paracetamol. It was noted that the adverse effect of microplastic in the presence of paracetamol was enhanced with respect to all studied parameters in test cyanobacteria.

**Fig. 2:**
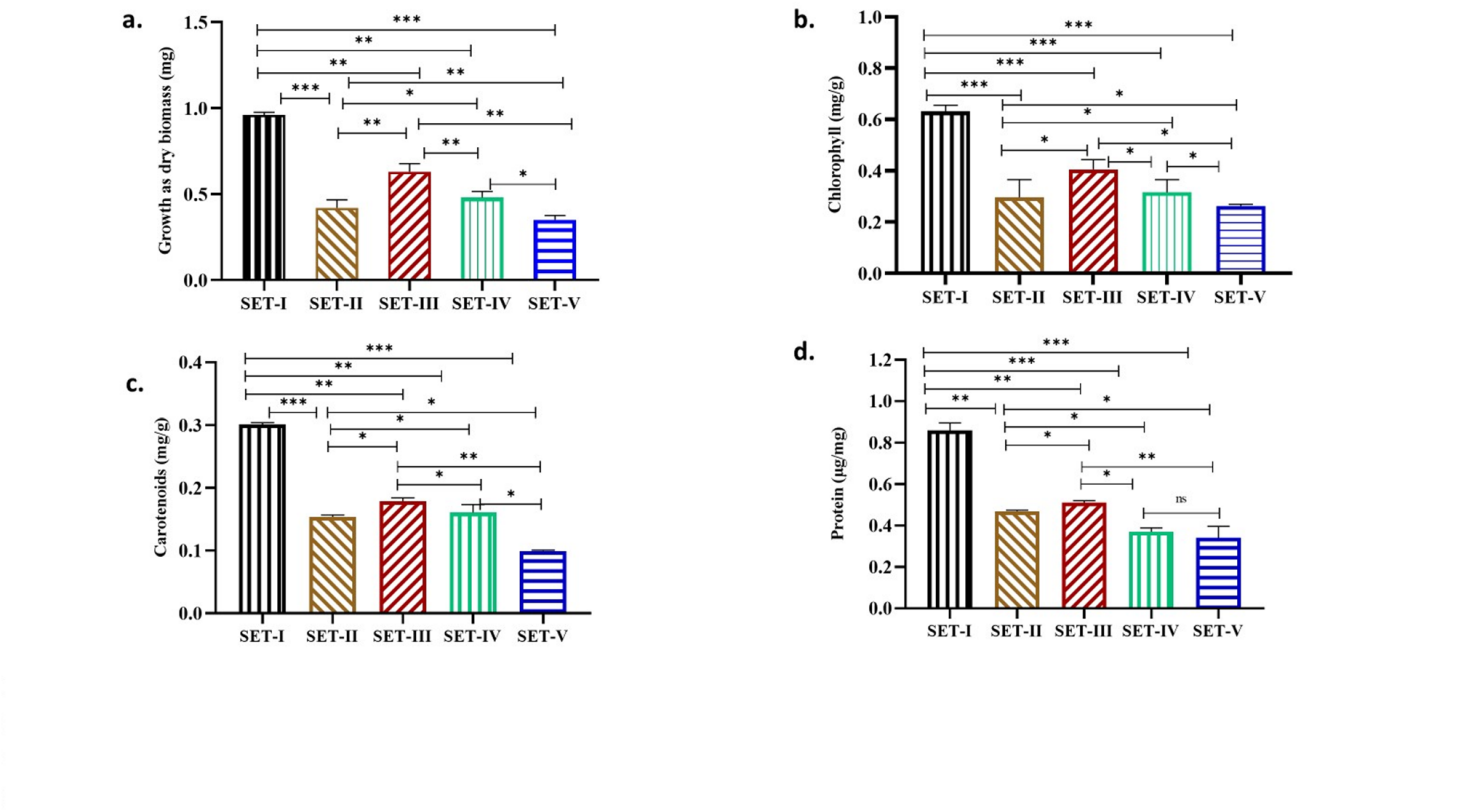
Effect of Acetone (AT), Polystyrene (PS), and Paracetamol (PCM) on the a.) growth (as dry biomass-mg) b) chlorophyll (mg/g), c) carotenoids (mg/g) and d) Protein (µg/mg) of Nostoc muscorum. SET-I (Control), SET-II (Control + Acetone), SET-III (Control + PS), SET-IV (Control + PCM), SET-V (Control + PS + PCM). All the values are the means of three replicates with standard deviation and asterisks (*) indicating the level of significance at *P* < 0.05, ns = not significant.

The growth of *Nostoc muscorum* was reduced to 56% in SET-II, 34% in SET-III, ) and 50% in SET-IV. The extent of growth reduction reached 63% after exposed to polystyrene spiked with paracetamol [(Fig. 2a SET-V), (significant at *P* ≤ 0.05)]. The higher toxicity in culture supplemented with PCM + PS, suggest that polystyrene provide an extra surface for adsorption and absorption of spiked pollutant. Polystyrene negatively affects the growth of photosynthetic organisms such as *Skeletonema costatum*^29^, *Euglena gracilis*^30^, *Microcystis aeruginosa*^31^ *Alexandrium pacificum*^32^, *Scenedesmus obliquus*^33^, and Like polystyrene, paracetamol also reduce the growth of aquatic organisms in a dose-dependent manner e.g., cyanobacteria *Nostoc muscorum*^34^, and green algae *Scenedesmus suspicious*^35^, *Chlorella ellipsoidea*^36^, *Chlorella vulgaris and Scenedesmus dimorphus*^37^. Spiking of MP with co-contamination studies are very recent and thus have very few examples could be found, especially of the photosynthetic organism but^38^ investigated that PS allowed the adsorption of pharmaceutical products -Acyclovir (an antiviral drug) in crustaceans- *Ceriodaphnia dubia*. that caused a higher inhibition of growth, reproduction and DNA damage.

### Effect of PS (pristine and spiked with PCM) on pigments and proteins of Nostoc muscorum

The pigments content (Chlorophyll & Carotenoids) of *Nostoc muscorum* decreased in all sets as compared to the control. The toxic effect of polystyrene on chlorophyll and carotenoid was less than paracetamol (Fig. 2b, c) SET III and SET IV respectively (significant at *P* ≤ 0.05). The maximum reduction in chlorophyll content (59%) was noticed in cultures exposed to polystyrene and paracetamol in combination i.e., after spiking (Fig. 2b, SET-V), whereas 59% reduction in culture exposed to acetone (Fig. 2b, SET-II), 44% in culture exposed to polystyrene (Fig. 2b, SET-III) and 55% with paracetamol (Fig. 2b, SET-IV).

Carotenoids, the culture supplemented with Polystyrene spiked with paracetamol also showed maximum reduction 69% as compared to the control (Fig. 2c, SET-V), whereas the culture exposed to acetone showed 48%, polystyrene exposed culture showed 40% (Fig. 2c, SET-III)) and paracetamol exposure exhibited 46% reduction (Fig. 2c, SET-IV). Less decrease in carotenoids in comparison to chlorophyll may be due to their role in stress tolerance.

The protein content also decreased in all treated sets than control (Fig. 2d). The culture SET-V showed maximum reduction 61% as compared to the control, whereas the culture SET-II, III, & IV showed 45%, 26% & 40% protein reduction respectively as compared to the control (significant at *P* ≤  0.05).

Higher reduction in photosynthetic pigment (chlorophyll & carotenoids) and protein was found in culture supplemented with polystyrene spiked with paracetamol, which indicated that PS increased the adsorption of paracetamol which in turn induced the disruption in the photosynthetic system and protein synthesis. Paracetamol-induced pigment and protein inhibition was also observed in *Nostoc muscorum*^34^, while PS-induced pigment and protein reduction was observed in *Chlorella ellipsoidea*^36^, *Pseudokirchneriella subcapitata*, *Scenedesmus dimorphus* and *Chlamydomonas reinhardtii*^37^, in *Chlamydomonas reinhardtii*^39^, in *Chlorella vulgaris*^40,41^, in *Acutodesmus obliquus*^42^ and *Platymonas helgolandica*^43^.

### Effect of PS (pristine and spiked with PCM) on MDA, H_2_O_2_ and electrolyte leakage of Nostoc muscorum

Biotic as well as abiotic stress led to the formation of free radicals i.e., reactive oxygen species (ROS) and reactive nitrogen species (RNS). These oxidative species are highly reactive in nature due to free electrons in their outer shell. These oxidative radicals trigger the chain reaction and make different kinds of oxidative radicals such as superoxide radicals, hydrogen peroxide, hydroxy radicals, per hydroxy radicals and alkoxy radicals^44^. In cyanobacteria, the main reason for the formation of free radicals is the inhibition of photosynthetic chain reaction in photosynthetic apparatus and membrane peroxidation that damage the membranal integrity and cause the leakage of electrolytes^45^.

**Fig. 3:**
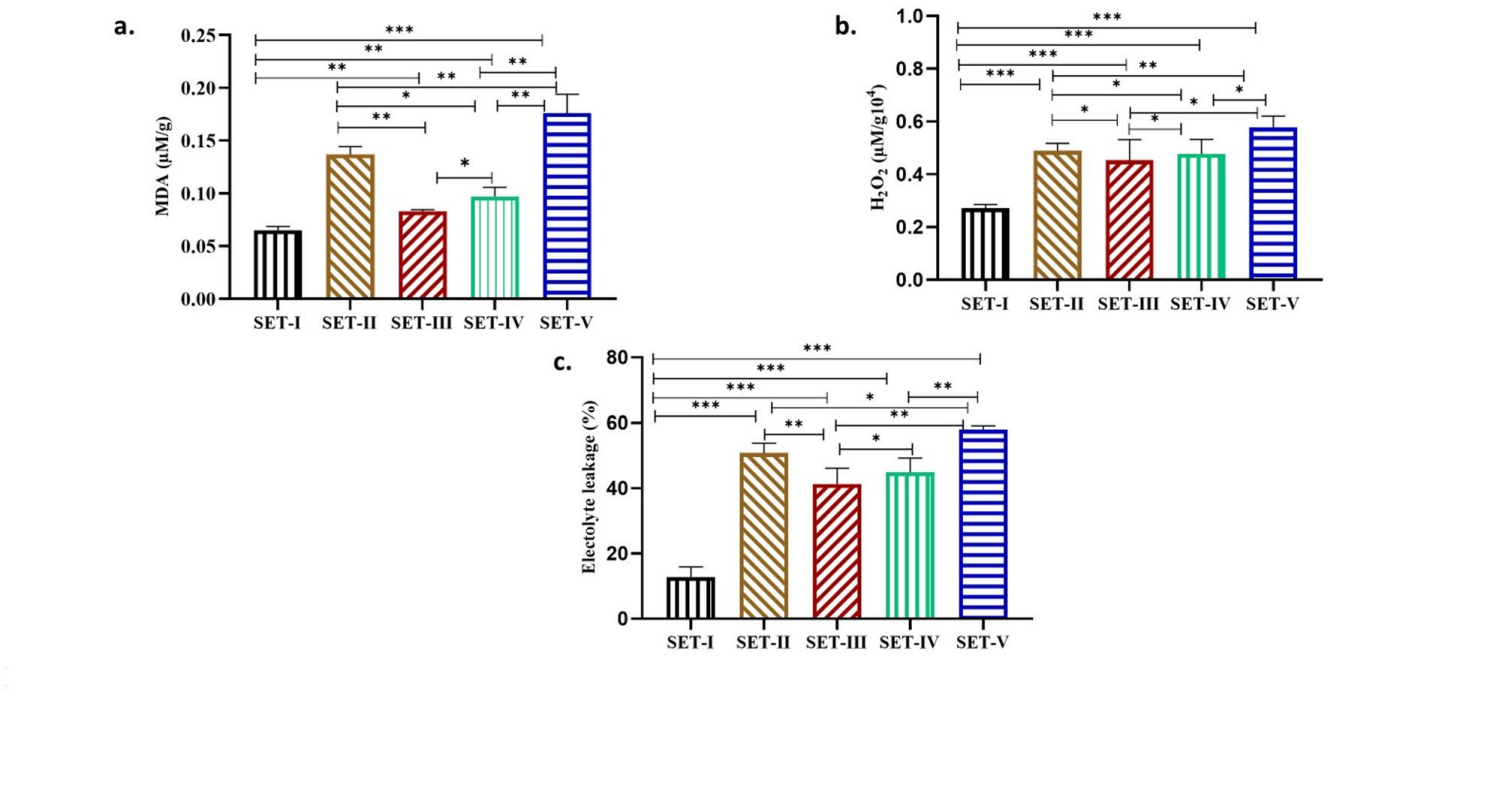
Effect of Acetone (AT), Polystyrene (PS), and Paracetamol (PCM) on the a.) MDA (µM/g) and c) H_2_O_2_ (µM/g) × 10^4^ and c) electrolyte leakage (%) of Nostoc muscorum. SET-I (Control), SET-II (Control + Acetone), SET-III (Control + PS), SET-IV (Control + PCM), SET-V (Control + PS + PCM). All the values are the means of three replicates with standard deviation and asterisks (*) indicating the level of significance at *P* < 0.05.

To find out the role of MDA in exerting microplastics (polystyrene) and paracetamol toxicity alone and in combination, a comparative study was performed. It was observed that the MDA content was increased in all sets as compared to the control (Fig. 3a). Maximum MDA content (58%) was noted in the culture with polystyrene spiked with paracetamol (Fig. 3a, SET-V). The culture with acetone, polystyrene and paracetamol alone showed only a 52%, 22% & 32% increase in MDA respectively [(Fig. 3a, SET-II, III & IV), (significant at *P* ≤ 0.05)].

Similarly, the level of hydrogen peroxide was also increased in all sets as compared to the control (Fig. 3b). Maximum hydrogen peroxide (52%) was found in the culture with both polystyrenes spiked with paracetamol (Fig. 3b SET-V). The culture with acetone, polystyrene and paracetamol alone showed only 45%, 40% & 43% hydrogen peroxide respectively [(Fig. 3b SET-II, III & IV), (significant at *P* ≤ 0.05)].

The increased level of MDA, and hydrogen peroxide in culture exposed to polystyrene spiked with paracetamol resulted in the highest membranal damage that caused increased electrolyte leakage (62%). (Fig. 3c SET-V). The culture with acetone, polystyrene and paracetamol alone showed lesser electrolyte leakage at 52%, 38% & 51% respectively [(Fig. 3c SET-II, III & IV), (significant at *P* ≤ 0.05)].

To the best of our knowledge, no study has been done on the effect of polystyrene spiked with paracetamol on *Nostoc muscorum*. However, polystyrene-induced increase in ROS production and membranal damage was studied in microalgae such as *Chlorella* and *Scenedesmus*^46^, *Synechococcus*^47^, *Chlamydomonas reinhardtii*^39^, *Chlorella vulgaris*^33,40^ and *Microcystis aeruginosa*^48^. Similarly, paracetamol-induced oxidative damage was reported in *Nostoc muscorum*^34^, *Lemna minor*^49,50^.

### Effect of PS (pristine and spiked with Paracetamol) on SOD, CAT, APX and proline of Nostoc muscorum

To quench the free radicals, cells increase the production of antioxidant molecules (enzymatic and non-enzymatic). Superoxide dismutase (SOD) enzymes provide the first line of defence to the organism but produce hydrogen peroxide (H_2_O_2_) as a by-product, which activates peroxidase enzymes such as catalase-peroxidase (CAT) and ascorbate peroxidase (APX) for the protection from the oxidative ions. To check the enzymatic antioxidant efficacy to quench polystyrene-induced free radicals in test organism SOD, CAT and APX activity was quantified.

**Fig. 4:**
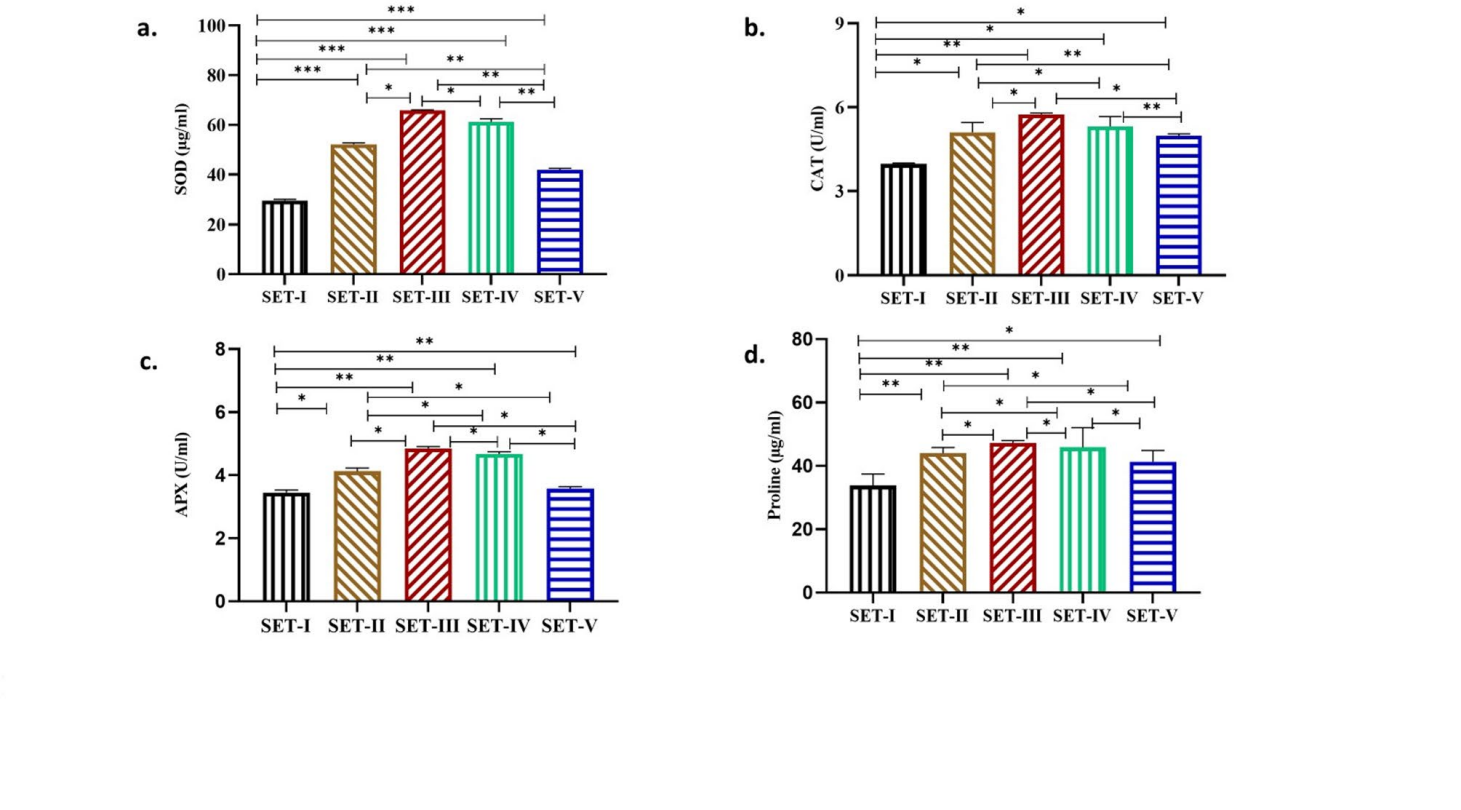
Effect of Acetone (AT), Polystyrene (PS), and Paracetamol (PCM) on the a.) SOD (µg/ml) and b) CAT (U/ml) c) APX (U/ml) and d) Proline (µg/ml) of Nostoc muscorum. SET-I (Control), SET-II (Control + Acetone), SET-III (Control + PS), SET-IV (Control + PCM), SET-V (Control + PS + PCM). All the values are the means of three replicates with standard deviation and asterisks (*) indicating the level of significance at *P* < 0.05.

The least increment of SOD activity was observed in culture exposed to polystyrene spiked with paracetamol 40% as compared to control (Fig. 4a, SET-V). Whereas the cultures exposed to acetone, polystyrene and paracetamol alone exhibited higher SOD activity (44%, 55% and 52%) respectively [Fig. 4a, SETII, SET III and IV, (significant at *P* ≤ 0.05)]

Similarly, among the treated culture minimum CAT activity (20%) was recorded in cultures with polystyrene spiked with paracetamol (Fig. 4b SET-V) as compared to control cultures (Fig. 4- b, SET-I). Whereas the cultures exposed to acetone, polystyrene and paracetamol separately exhibited higher CAT activity (22%, 31% and 26%) respectively [Fig. 4b, SET-II, SET III and IV, (significant at *P* ≤ 0.05)].

The culture with polystyrene spiked with paracetamol showed lowest APX activity (16.2%) while acetone, polystyrene and paracetamol separately exhibited (30%, 36% & 34%) of APX activity respectively [Fig. 4-c, SET-II, SET-III & IV, (significant at *P* ≤ 0.05)].

The SOD, CAT, APX data suggested increased susceptibility to oxidative stress in *Nostoc muscorum* when exposed to microplastic was present with another pollutant like paracetamol.

To our best knowledge, no report is available on antioxidant enzymes MP spiked with the co-contaminant photosynthetic organism and our effort represents the novelty of the work. However^51^, also reported that polystyrene increased the activity of antioxidant enzymes in *Scenedesmus obliquus*. The green algae such as *Chlorella vulgari*^33^ and *Chlorella pyrenoidosa*^52^, *Euglena gracilis*^30^ also activate the antioxidant defence response by increasing the antioxidant enzymes on exposure to polystyrene. Paracetamol also induced CAT and APX antioxidants activities in aquatic *Nostoc muscorum*^34^ and *Lemna minor*^49^ as well as terrestrial plant *Brassica juncea*^53^.

In treated culture the proline content found was minimal in cultures with polystyrene spiked with paracetamol (18%) (Fig. 4d SET-V) as compared to control cultures (Fig. 4- d, SET-I). Whereas the cultures exposed to acetone, polystyrene and paracetamol individually exhibited higher proline content (23%, 28% and 26%) respectively (Fig. 4d, SET III and IV). The higher toxicity in SET-V may be due to the availability of less proline (significant at *P* ≤ 0.05). A non-enzymatic antioxidant such as proline acts as an osmoprotectant, maintaining osmolality and protecting the cell from oxidative bursts under stressful conditions. Similarly, in mung bean, polystyrene alone and spiked with lead increased the proline content^54^.

### Effect of PS (pristine and spiked with Paracetamol) on activity of degrading enzymes (Laccase & Esterase) of Nostoc muscorum

**Fig. 5:**
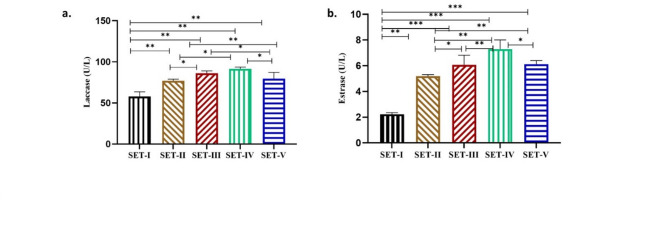
Effect of Acetone (AT), Polystyrene (PS), and Paracetamol (PCM) on the (a) Laccase (U/L) and (b) Esterase (U/L) of *Nostoc muscorum*. SET-I (Control), SET-II (Control + Acetone), SET-III (Control + PS), SET-IV (Control + PCM), SET-V (Control + PS + PCM). All the values are the means of three replicates with standard deviation and asterisks (*) indicating the level of significance at *P* < 0.05.

Laccase activity was higher in SET-IV (37%) whereas the culture SET-II, SET-III and SET-V showed less increase in laccase activity (25%, 33% and 27%, respectively) (Fig. 5a). Similarly, maximum esterase activity was recorded in culture SET-IV (69%) and minimum in SET-II (56%) as compared to control (Fig. 5b). The culture SET-III and SET-V showed 66% and 64% increase in esterase activity.

The cleavage of polymer chains into monomers and oligomers occurs during the biodegradation of microplastics due to the action of certain enzymes in cyanobacteria such as laccase and esterase^55^. Further, these monomers and oligomers are absorbed by cells to be metabolized^56^. Since cyanobacteria are aerobic bacteria, therefore, carbon dioxide and water are produced as an end product that is further used by cells for photosynthetic activity. The increased activity of laccase and esterase in *Nostoc muscorum* also indicated that the cells may break down and metabolize polystyrene.

The two aerobic organisms *Aspergillus oryzae* and *Bacillus cereus* also reported increased laccase and esterase activity after exposure to low-density polyethene plastic^57^.

### Confocal microscopy of PS pristine and spiked of Nostoc muscorum

**Fig. 6:**
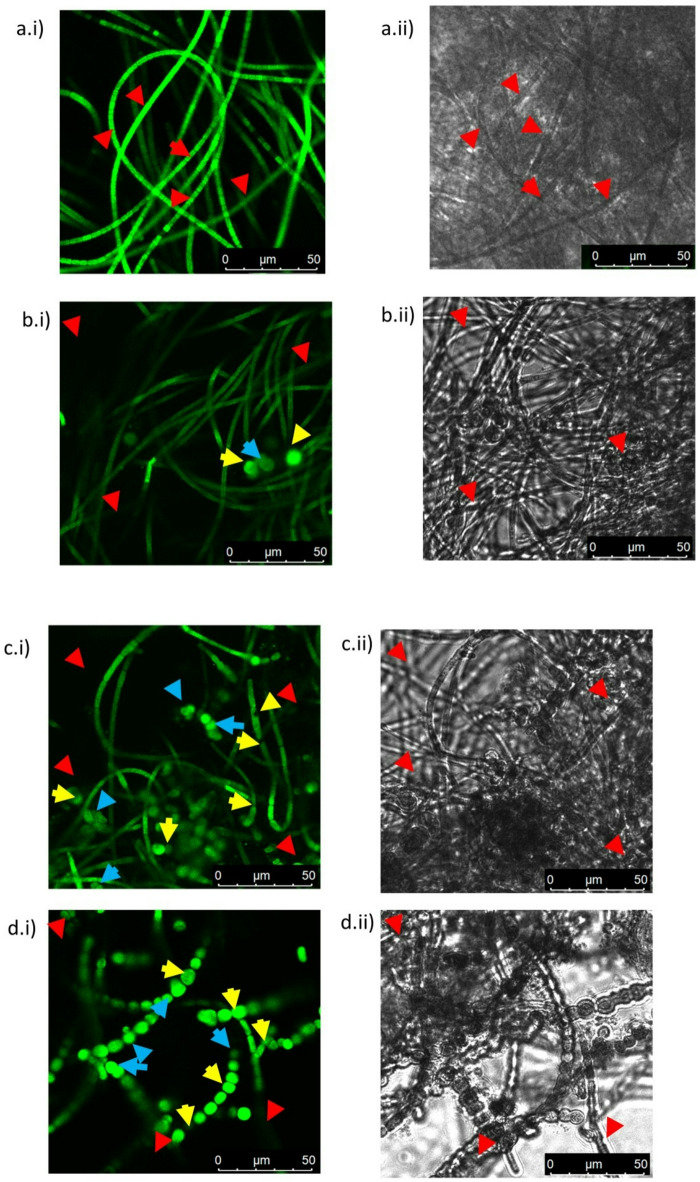

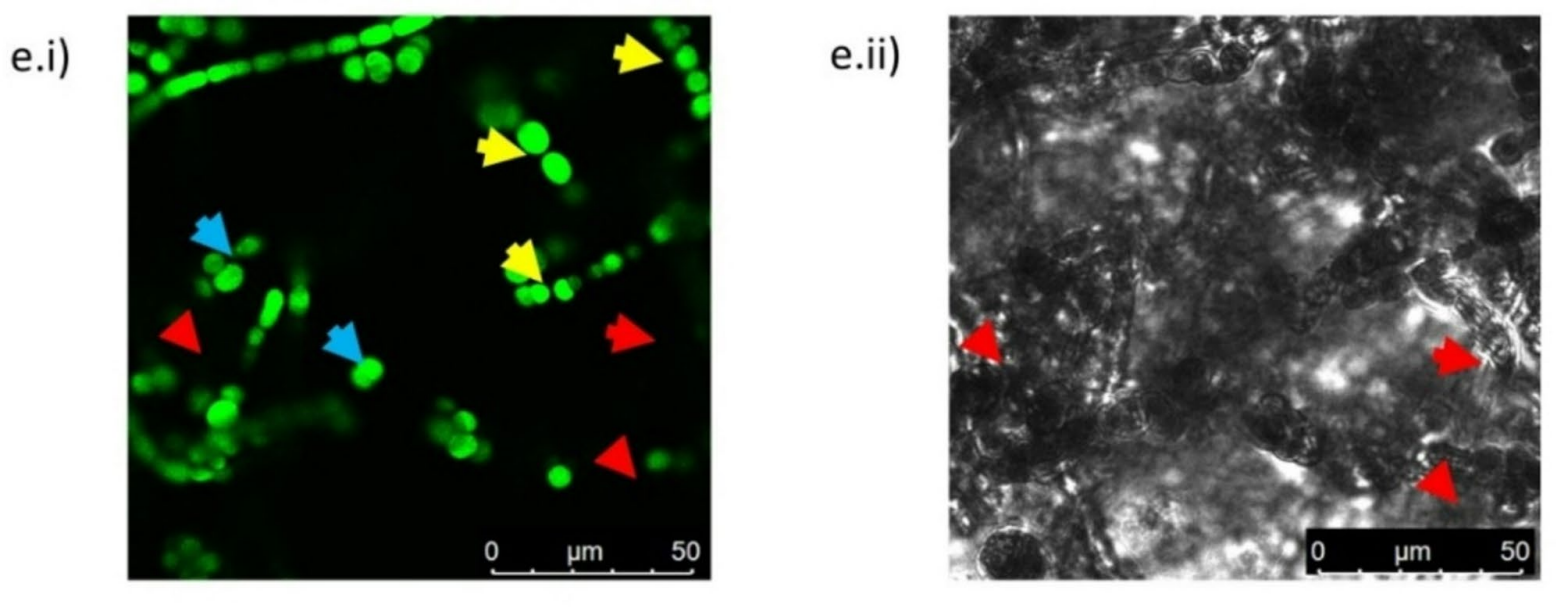
(a–e) Confocal microscopy of Nostoc muscorum taken from SET-I, SET-II, SET-III, SET-IV, & SET-V; a.i.), bi), ci), di) & ei) are fluorescent images and aii), bii), cii), dii) & eii) are phase contrast images. Red arrow indicates the cells in both side, yellow arrow indicates cell fragmentation and blue arrow indicate cell division.

After finding biochemical evidences of PS (pristine & spiked with PCM) toxicity in *Nostoc muscorum* an effort was made to examine alteration in cyanobacterial growth by confocal studies. By measuring fluorescence intensities of confocal images of the samples that decreased with increasing toxic effect of polystyrene when spiked with paracetamol (Fig. 6a–e). Maximum fluorescence and filamentous nature of *Nostoc muscorum* were evident in control (SET –I) representing best growth. Fluorescence was least in cells exposed to acetone (SET II) that was used as Polystyrene solvent in further sets. The sample exposed to polystyrene showed higher fluorescence (SET III) than acetone (SET II). This may be due to availability of additional carbon supply by the polystyrene breakdown. Paracetamol exerted higher toxicity on *Nostoc muscorum* (SET IV) than polystyrene (SET III) in terms of loss of filaments. Probably the cells counteracted the toxicity by changing the morphology to rounded shaped. Polystyrene spiked with paracetamol exhibited higher toxicity (SET V) than polystyrene alone (SET III) and paracetamol (SET IV). The rounding of the cells (resting phase) was more evident under the combined effect. The resting stage reduces growth.

### Scanning electron microscopy of PS pristine and PCM spiked of Nostoc muscorum

**Fig. 7:**
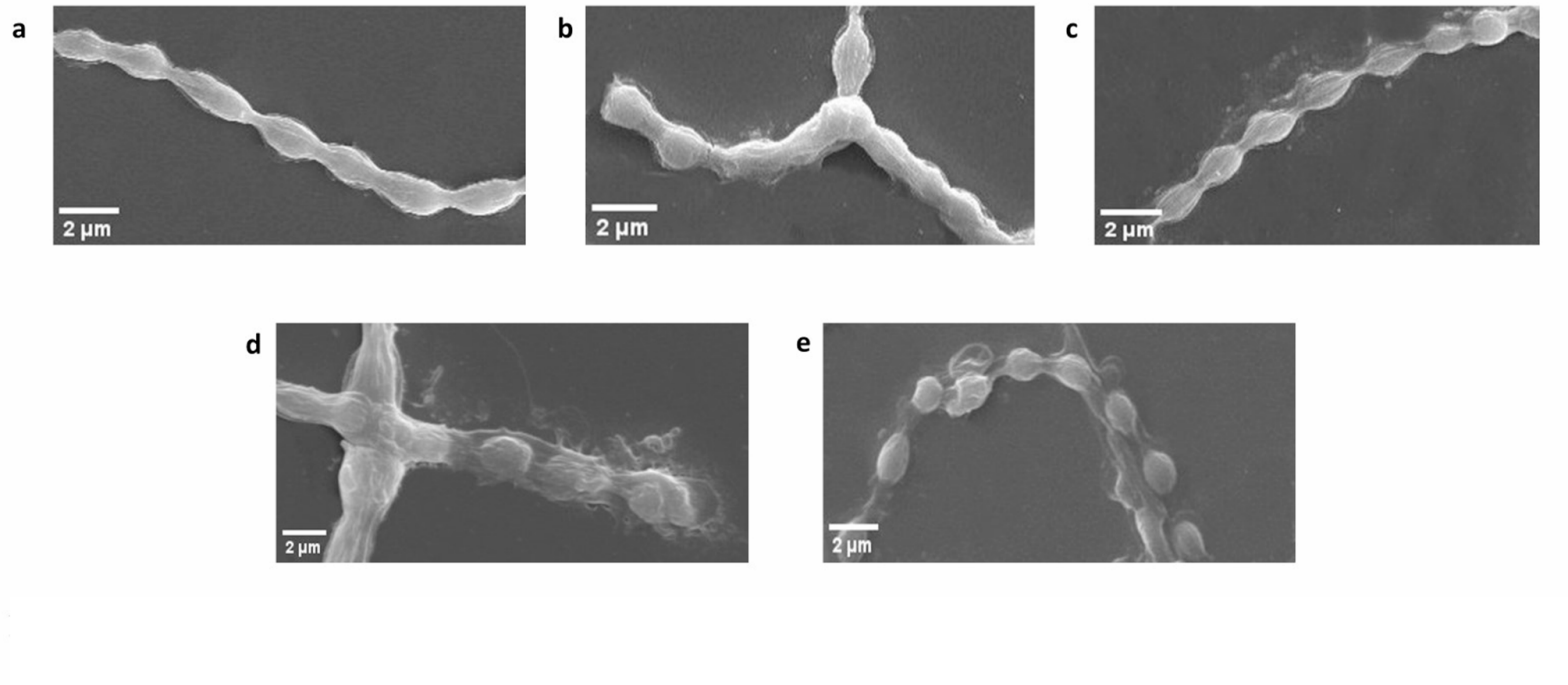
(a–e) Scanning electron microscopy of *Nostoc muscorum* taken from SET-I, SET-II, SET-III, SET-IV, & SET-V; a is control, b is AT treated, c is PS treated, d is PCM treated & e is AT + PS + PCM treated cells.

Scanning electron micrographs showed a significant difference among all experimental sets (Fig. 7a–e). After 24 days of growth, SET –I (Control) cells were healthy and exhibited normal cell division. The SEM images obtained from rest sets (SET-II, SET-III, SET-IV and SET- V) showed cell distortion and fragmentation as compared to the control. Maximum cell distortion and fragmentation were seen in SET-V (PS spiked with PCM). It was also associated with cell lysis. Similarly, cellular distortion was also observed in *Nostoc muscorum* Meg 1 under herbicide stress^58^ and in *Nostoc flagelliform* under UV stress^59^.

### Transmission electron microscopy of PS pristine and PCM spiked of Nostoc muscorum

**Fig. 8:**
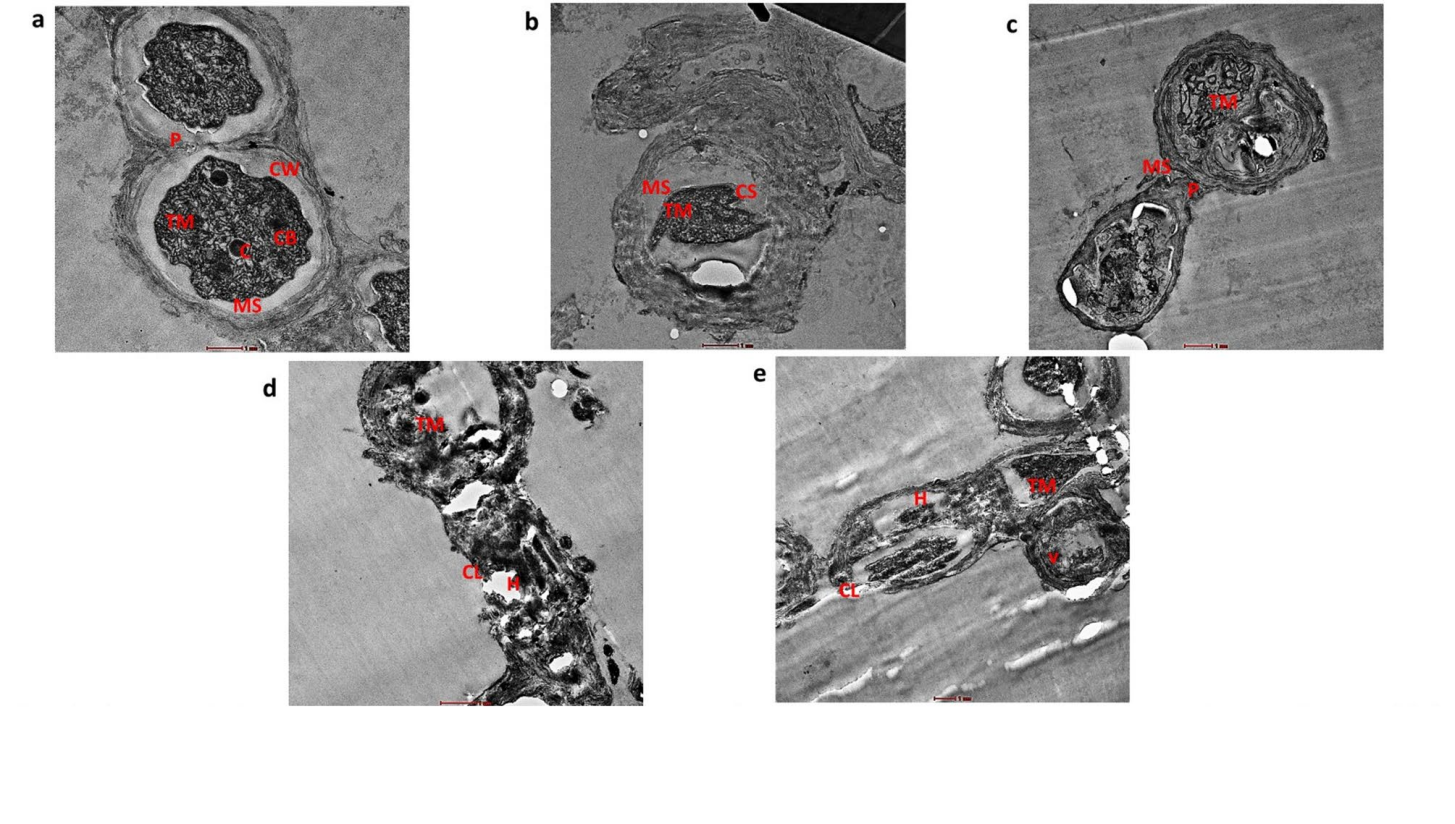
(a–e) Transmission electron microscopy of Nostoc muscorum taken from SET-I, SET-II, SET-III, SET-IV, & SET-V; a is control, b is AT treated, c is PS treated, d is PCM treated & e is AT + PS + PCM treated cells. CW = cell wall, TM = thylakoid membrane, CB = carboxysomes, C = cyanophycean granules, MS = mucilaginous sheath, P = pore, V = vacuole, H = heterocyst, CS = cell shrinkage, CL = cell lysis.

In confocal and scanning electron micrographs, cellular alteration was observed. The internal alteration in the exposed cells transmission electron microscopy was done for further analysis (Fig. 8a–e). Most cellular components such as thylakoid membrane, carboxysomes, cyanophycean granules, pores, and mucilaginous sheath are visible in control and SET-III. SET-II and SET III culture cells showed cell shrinkage and deformed thylakoid cells. In SET-IV and SET-V culture cells, the disintegration of the cell membrane and thylakoid membrane was observed. The mucilaginous sheath was disintegrated in SET-II, SET-III, SET-IV and SET-V culture cells. Similarly, internal damage was also observed in *Nostoc carneum*, *Planktothrix cryp-tovaginata*, *Microcystis aeruginosa*, and *Scenedesmus acutus* under UV-B stress^60^.

## Conclusion

The present work highlighted the interaction of polystyrene (microplastics) with co-contaminate paracetamol (a nonsteroidal anti-inflammatory drug) in a cyanobacterium- *Nostoc muscorum*. The results showed that the polystyrene spiked with paracetamol exhibited a higher adverse effect on the growth and biochemical constituents than their pristine forms.

The results showed that PS acts as a vector or carrier of other pollutants that are present in aquatic bodies and is becoming a great threat to the present and future generation’s health and existence.

## Data Availability

The datasets used and/or analysed during the current study available from the corresponding author on reasonable request.
